# Ethical challenges of integration across primary and secondary care: a qualitative and normative analysis

**DOI:** 10.1186/s12910-019-0386-6

**Published:** 2019-07-03

**Authors:** Alex McKeown, Charlotte Cliffe, Arun Arora, Ann Griffin

**Affiliations:** 1 Department of Psychiatry, University of Oxford, Warneford Lane, Oxford, OX3 7JX England; 2 University College London Medical School, 74 Huntley Street, London, WC1E 6AU England; 3 University of Manchester Medical School, Oxford Road, Manchester, M13 9PL UK; 4 Research Department for Medical Education, University College London Medical School, 74 Huntley Street, London, WC1E 6AU England

## Abstract

**Background:**

This paper explores ethical concerns arising in healthcare integration. We argue that integration is necessary imperative for meeting contemporary and future healthcare challenges, a far stronger evidence base for the conditions of its effectiveness is required. In particular, given the increasing emphasis at the policy level for the entire healthcare infrastructure to become better integrated, our analysis of the ethical challenges that follow from the logic of integration itself is timely and important and has hitherto received insufficient attention.

**Methods:**

We evaluated an educational intervention which aims to improve child health outcomes by making transitions between primary to secondary care more efficient, ensuring children and parents are better supported throughout. The programme provided skills for trainee paediatricians and general practitioners (GPs) in co-designing integrated clinical services.

**Results:**

The key ethical challenges of integrated care that arose from a clinical perspective are: professional identity and autonomy in an integrated working environment; the concomitant extent of professional responsibility in such an environment; and the urgent need for more evidence to be produced on which strategies for integrating at scale can be based.

**Conclusions:**

From our analysis we suggest a tentative way forward, viewed from a normative position broadly situated at the intersection of deontology and care ethics. We adopt this position because the primary clinical ethical issues in the context of integrated care concern: how to ensure that all duties of care to individual patients are met in a newly orientated working environment where clinical responsibility may be ambiguous; and the need to orientate care around the patient by foregrounding their autonomous preferences and ensuring good patient clinician relationships in clinical decision-making.

**Electronic supplementary material:**

The online version of this article (10.1186/s12910-019-0386-6) contains supplementary material, which is available to authorized users.

## Background

Arguments for integration have been well articulated, for example in the UK government's landmark NHS Five Year Forward View [1]. Moreover, healthcare funding is a source of profound public controversy in the UK [2]. Whether the escalating shortfall in provision [3] is due to under-investment by government for political reasons [4, 5] or poor workforce planning [6], is not a matter for judgement here. However, the case for integration is frequently made against this backdrop to ensure that available resources are used optimally effectively.

The term ‘integration’ has a general conceptual meaning and a more specific contextual meaning in healthcare. Broadly speaking, to integrate means to merge parts into a whole [7], and this is a reasonable description of the *goal* of care integration. However, in this context, integration also refers to the *mechanisms* by which integration can occur [8]. Healthcare integration aims to connect discrete ‘silos’ of disciplinary expertise to reduce gaps in care so patients, or in the case of our study, children and parents or carers, are supported seamlessly throughout treatment [9, 10], including overcoming barriers between generalist and specialist care [11]. Integration draws on the perspectives of those receiving care to orientate its provision around their needs [9, 12, 13] through shared decision-making with the medical professionals. In the context of paediatrics, Wolfe et al (2011, p. 992) [14] define integrated care as follows:

*‘ … health services organised and managed so that people get the care they need, when they need it, in ways that are user-friendly, achieve the desired results and provide value for money … Integrated care means … better configuring services within healthcare and between health and other sectors’*

In recent years in UK healthcare there has been increasingly familiar call to ‘break down silos’ [15] and to remove barriers between specialist areas for more effective collaboration [16]. The ultimate aim to provide a healthcare system that is holistic, efficient, and seamless. It is therefore important to understand why achieving this aim is complex and challenging [17].

The normative anchor for integration is the duty to deliver optimal care by harmonising the tasks of the specialist professionals involved and the domains of expertise in which they have autonomy [18]. Ethical and economic cases can be advanced in favour of this. If improving efficiency will provide better care for more people, then we *ought* to aim to achieve this [10, 11]. This is the straightforward ethical case. The economic case is predicated on the growth in chronic diseases exerting significant pressures on healthcare resources [19, 20]^1^. Our study focused on integrated care for children where the burden of chronic disease has seen a massive growth in recent years, with estimates of 13-27% of the childhood population being affected [21].

It is also significant that we live in an era of increasing specialisation in medicine and healthcare [22, 23]. It has been argued that there is a need to recover the ‘whole patient’ from the diffuse systems across which their health problems have been spread [24], to achieve a more sophisticated form of generalism [25] that can *integrate* these specialist domains in the holistic interest of the recipient. An approach that uses the patient’s experience in designing care is thus vital. It helps clinicians, commissioners, and policy-makers gain insight into gaps or inefficiencies that are experienced by the patient towards improving their journey through the system [9, 13].

Pilot studies in integration at local and regional levels continue to emerge both in the UK [26–28] and elsewhere [29–31]. Moreover, given their well-established history there is robust guidance for how to develop Integrated Care Pathways, which formalise stages of patient progression for specific conditions [32–34]. While it is true, given their now well-established history, that considerable evidence and data exist for reliably establishing pathways for conditions with a well-known progression [35–37], less exists for system-wide integration *per se*. It is the ethical challenges that follow from achieving this that we consider here.

### Theoretical accounts of integration

**Fig. 1:**
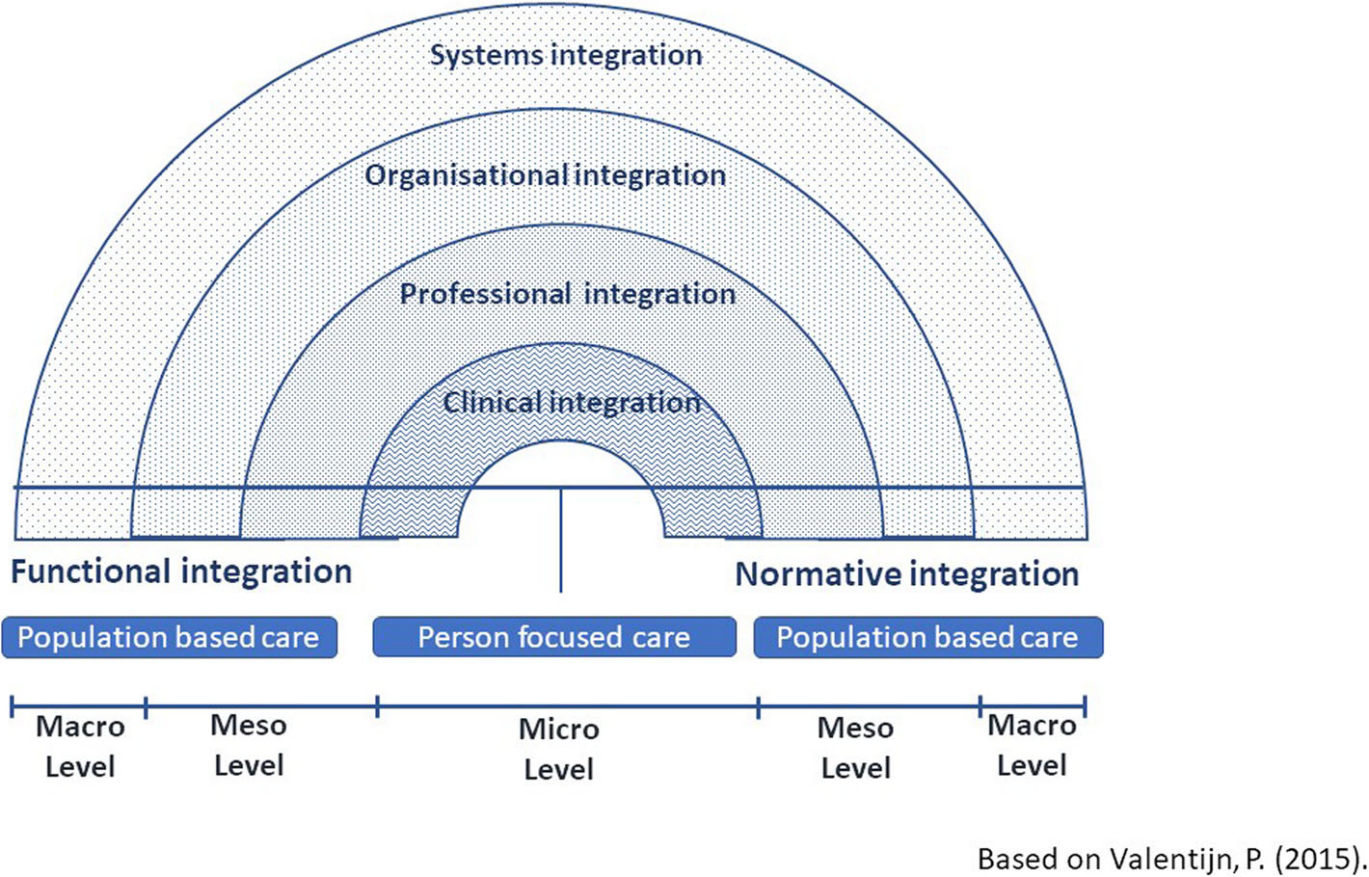
The Rainbow Model of Intergrated Care

The Rainbow Model of Integrated Care (RMIC) [38] (see figure 1) is a common theoretical characterisation of how system-wide integration might be realised. The model uses concentric semi-circular rings to show the different scales at which integration must occur and how integration must radiate out from the patient, to the clinical team, embedded in an organisation, operating as part of a larger system. Nevertheless, the concentric rings which separate these remain, and their presence underlines the difficulty that integration wishes to overcome, namely, naturally occurring delineations between domains of expertise.

Similarly, Suter et al. [39] posit ten key principles for successful healthcare integration namely: continuous care; patient focus; geographic covering; standardised care in interdisciplinary teams; performance management; information systems; organisational culture; physical integration; governance; and financial management. Undoubtedly these *are* important, but they are similarly aspirational and fall short of engaging with the ethical challenge of how *in practice* integration is to be negotiated.

We contend that despite a range of theoretical models of how care *should* be integrated, the practicalities of doing so raise complications that pose fundamental ethical challenges which have hitherto not been sufficiently addressed. Indeed, the relative lack of a unified, shared, widely applicable approach [40] and empirical framework [41] implies support for our central claim, namely that greater investment is needed in the production of a relevant evidence base.

The final point of note here is that integrated care is, arguably, consistent with the general direction of travel away from medical paternalism towards a norm in which good care is understood as giving *equal* weight to patients’ autonomous wishes [42–44] and a clinician’s professional judgement. The co-design aspect of integrated care is thus congruent with both the economic and the ethical arms of the arguments in favour of it [45, 46].

### Ethical Perspectives on Integrated Care

Several approaches in ethics could frame the challenges we identify, and some of these are more helpful than others. Different normative orientations may produce different, and sometimes conflicting, accounts of what ought to be valued and done. For reasons we summarise, the approach we adopt exists at the intersection of deontological and care ethics approaches. Our analysis is, in turn, situated more broadly in the critical realist paradigm with respect to the integration of the empirical and normative components. In grounding events and perspectives thereon in a shared external reality, critical realism provides the conditions for intersubjective agreement, and is therefore useful for ethical analysis which draws on empirical findings. Space forbids a fuller methodological account of how the integration of empirical and normative components can be achieved using critical realism, but such an account has been provided by McKeown (2017) [47].

We understand our position as broadly deontological because our concern in evaluating integrated care is how it helps or hinders clinicians to discharge their duties to specific individuals. This is not to say that an ethics of integrated care is interested in the discharging of duties *alone* and *un*interested in whether or not this produces the desired consequences, since clearly clinicians want patients to be better after treatment than they were before it [48]. Rather, what is important to a deontological view is that individuals may not be used only as means to the realization of an end defined as good by the *amount* of people who stand to benefit [49]. Although justice concerns at the macro- scale are pertinent to questions of optimally efficient resource allocation, our primary concern is to highlight potential risks to individual patients. As such, broader, political, questions of distributive justice relating to resource allocation, while important, are orthogonal to our analysis, rather than central to it.

We combine our broadly deontological outlook with care ethics, which is consistent with what we take to be the principles of integrated care. Care ethics foregrounds the importance of understanding what is contingently needed and considered important by the care recipient, by cultivating strong, empathetic relationships between patients and clinicians [50], irrespective of any particular moral theoretical orientation [51, 52]. Its normative grounding turns on what one should do given *the particular needs of a particular individual in a particular clinical context*.

We note criticisms of care ethics, however. For example, care ethics has been a subject of feminist critique because, as Keller (1997, p. 153) [53] points out, since *‘caring is inculcated in girls and women through socialization processes that curb their ambitions and abilities’,* so our assumptions about care and who should perform it have a tendency to undermine female autonomy and reinforce pernicious traditional gender roles [54, 55]. This criticism may be reasonable in a general sense, but we doubt that it functions as a serious criticism of paediatrics, given that the balance between female and male paedatricians is 52 / 48% respectively in the UK [56] and 73 / 63% in the USA [57].

Similarly, in responding to the criticism that care ethics considered as ‘an’ ethics is empty, given the vagueness of an edict that states simply that people should care for each other, Verkerk (2001, p.290) [58] contends that its value derives from it being seen as *‘an orientation’* or *‘a perspective’* that aims towards *‘living good in concrete relationships with others, responding to their needs and building up a joint life’*. This pragmatic and person-centred view is consistent with principles of integrated care outlined in relevant guidance of the kind identified earlier, and as such that it cannot be understood as a moral *theory* does not count against its adequacy for our purposes.

In light of these rebuttals of the criticisms levelled at care ethics, therefore, we contend that it can be used to broadly characterise the key normative principle of integration; namely, that care should be patient-centred and foreground the preferences of individuals in the varied and unpredictable clinical settings in which they find themselves and in which they are encountered by clinicians.

What we have sketched out is necessarily a simplified exegesis of a complex, inter-related, body of literature and thought. To summarise our orientation regarding the ethical issues analysed, however, we can say the following. Our ethical standpoint is situated at the intersection of two approaches – one explicitly theoretical, one practically grounded. The theoretical component is a broadly deontological view, giving priority to clinical ethical duties to specific individuals, irrespective either of wider resource allocation dilemmas or the various ways in which principles such as beneficence and non-maleficence may be set against each other. The practical component derives from care ethics, in view of the importance that it places on good relationships that ensure patient needs and preferences are foregroumded, such that care is patient-orientated, irrespective of wider moral theoretical views or conflicts.

Although the theoretical component of our position is best described as deontological, it is not doctrinaire: we recognize the practical ethical implications of all moral theoretical approaches and this framing is therefore heuristic rather than didactic. We have sketched it out only for the purposes of roughly situating our analysis in the normative landscape. In our view, this heuristic framing is complemented by the practical care ethics component, given that the characteristic unpredictability of contexts in which care decisions must be made is one of the challenges that successful integrated care must be equipped to accommodate.

### Context

Our analysis follows from an evaluation carried out by the Research Department of Medical Education (RDME) UCL Medical School (UCLMS) for Health Education England (HEE) over twelve months from March 2016, of an integrated care education programme aimed to develop junior doctors’ skills in establishing integrated and person-centred care [59]. The programme provides a year-long programme for trainee paediatricians and GPs, including development of joint projects integrating services across primary and secondary care. These projects are supported by mentoring from senior clinicians with integrated care expertise, and monthly seminars where trainees can share learning and receive feedback.

The primary study evaluated the programme and provided an examination of doctor’s perceptions of integrated care and issues for implementation [60]. What follows is a secondary analysis of data foregrounding the ethical issues associated with integrated care that emerged in the context of these interviews.

## Methods

We employed an interpretive qualitative methodology, interviewing paediatric and general practice trainees and their mentors. Qualitative research permitted the exploration of issues central to integrated care. We used one-to-one interviews to understand participants views about the concept of integrated care, its aims, and its importance and the issues involved in delivering integrated care.

### Sample

The study population comprised paediatric trainees, GPs and mentors from the first and second programme cohorts and was set in London. Participants were invited to take part via the programme administrative team. Two reminder invitations were sent to those who had not responded to the initial request to take part in interviews. Table 1 provides a breakdown of the 23 participants who took part in the study.

**Table 1:** Table of study participants

Participant type: Trainee (T)/mentor(M)	Medical specialism: General practice (GP)/Paediatrician (P)	Cohort number:1 = 1st year 2 = 2nd year	Participant identifier
T	GP	Cohort 2	P1TGP2*
M	GP	Cohort 1 & 2	P2MGP
T	Paediatrician	Cohort 1	P3TP1
T	Paediatrician	Cohort 2	P4TP2
M	Paediatrician	Cohort 1 & 2	P5MP
T	Paediatrician	Cohort 2	P6TP2
M	Paediatrician	Cohort 1 & 2	P7MP
M	Paediatrician	Cohort 1 & 2	P8MP
T	Paediatrician	Cohort 2	P9TP2
T	Paediatrician	Cohort 1	P10TP1
M	Paediatrician	Cohort 1 & 2	P11MP
T	Paediatrician	Cohort 2	P12TP2
T	Paediatrician	Cohort 2	P13TP2
M	GP	Cohort 1 & 2	P14MGP
T	GP	Cohort 2	P15TGP2
M	Paediatrician	Cohort 1 & 2	P16MP
T	Paediatrician	Cohort 2	P17TP2
M	Paediatrician	Cohort 1 & 2	P18MP
T	GP	Cohort 2	P19TGP2
T	Paediatrician	Cohort 1	P20TP1
T	Paediatrician	Cohort 1	P21TP2
T	Paediatrician	Cohort 2	P22TP2
T	Paediatrician	Cohort 2	P23TP2

### Data gathering

Semi-structured interview schedules were developed by a postdoctoral researcher (AM) and the Principal Investigator of the study (AG) to evaluate the programmes aims. They were designed purposefully to explore issues identified in the literature about integrated care (See Additional file 1). Semi-structured schedules allowed the research team to explore lines of the enquiry and concepts deemed important to the study, while also allowing sufficient flexibility to allow respondents to contribute their views. Schedules were tested in a pilot study of four participants and only minor amendments were made. Interviews were conducted by all authors, either in person or by telephone, according to the interviewee’s preferences, audio recorded for accuracy and transcribed professionally, and lasted for between 20 and 60 minutes.

### Data analysis

Interview transcripts were analysed thematically. The interviews were independently coded by four researchers (AG, AM, CC, AA) using QSR NVIVO 11©. An initial coding scheme was developed based on analysing five transcripts. Each of the four team members coded the same five transcripts, and comparison and discussion thereof were used to devise the first iteration of the coding framework. Thereafter, the remaining 14 transcripts were distributed between three researchers for coding (AM, AA, CC). After this second round of coding, the analyses were compared and tested for inter-coder reliability. Remaining discrepancies were discussed to produce a final version.

### Ethical Approval

The study (8949/001) was given ethics clearance by the UCL Joint Research Office. Participants volunteered to take part and actively consented after having received a participant information sheet. All materials were anonymised and are held confidentially in compliance with the UK Data Protection Act 1998.

## Results

We present three themes of key ethical relevance to practicing integrated care that emerged from our data. These pertain to: professional identity and autonomy; responsibility; and expertise and evidence. Each entails issues of ethical concern which we raise and discuss.

### Professional Identity and Autonomy

The first theme related to the impact of integration upon the scope of a clinician’s autonomy. To the extent that professional autonomy is constitutive of professional identity, integration has an impact upon professional identity insofar as it poses a challenge to clearly delineated domains of medical jurisdiction. Our study found frequently that the clinicians believed integration and the changes to practice that it entails to be desirable and necessary, implying an ideal of professional identity that is collaborative, rather than separated or rigidly defined. However, since the programme pioneers a new approach to care, there were suggestions that the changes required to realise this may be difficult for some to accept:

*'I think people chose their speciality as a bit of a sort of … you know they kind of live the identity. So if you’re telling a consultant that maybe he should spend more of his time hanging out in people’s homes or in children’s centres or something like that – not quite got the status of you know a hospital consultant, you know that kind of thing. GPs I think don’t like having their patch trampled on.' (P7MP)*

Uncertainty thus emerged about what would incentivise doctors to adopt a new way of working in which their autonomy is curtailed and their professional identity made ambiguous:

*'...what’s the benefit, what’s the lever for a consultant paediatrician, who doesn’t know too much about integrated care, what would the lever be, what would make it better for them? (P16MP)*

Several participants were sceptical about the willingness for change across the whole healthcare infrastructure, stating that there is '*a culture'* of working independently and there are *'different agendas...as to how to deliver care' (P13TP2)*.

These comments indicate a challenge that can be hidden by the rhetorical power of the claim that more collaboration necessarily entails better care.

### Responsibility

The second theme is the corollary of autonomy; namely, responsibility. On several occasions, mentors highlighted challenges to integration where clinical responsibility has to be managed across specialist domains. For example, conventional ways of working in which responsibility for a patient is clearly situated in a particular clinical domain may be unsuitable to meet the needs of patients whose needs straddle specialist boundaries.

*'...children with complex needs that are predominantly under the care of the paediatrician and then as they become adults, adolescents … they’re then suddenly discharged...all of a sudden they become the full responsibility of the GP, having never made contact with the GP before because paediatric services are so good...it is almost a bit unfair for a GP to have to take on board a complex child or complex young adult who they’ve never met before, know nothing about...that is quite a grey area, they are formally under the care of the paediatrician, so they don’t need to see the GPs. As such the GPs become deskilled with dealing with their complex issues, and then all of a sudden they’re no longer part of paediatrics. So I don’t think it’s straight forward at all' (P7MP)*

In other cases the challenge came from an unwillingness to embrace new ways of working that would mean negotiating shared clinical responsibility, rather than continuing to assume traditional line of responsibility being ‘*compartmentalised’ (P15TGP2)* within a specialist/generalist domain. However, participants in this programme were mindful that this boundaried way of working was no longer legitimate.

‘ … *nobody comes in with a silo of problems. And in today’s NHS everything is run by … or should be run by a multi-professional team, because those are the resources we have. You can’t manage a patient as a single speciality on your own without ... not just your other colleagues in different specialities as medics, but also all of your other health care professionals, so your physios, your OTs, nurses and everything (P14MGP)*

### Expertise and Evidence

Although the programme is at the vanguard of integrated care education, several participants pointed out that it is the exception rather than the norm, and that integrated working occurs only inconsistently within the healthcare infrastructure. This led to the third theme more expertise and evidence of how integrated care can be delivered, such it meets the improved ethical standard that it is claimed to be capable of delivering. One participant highlighted the scarcity of clinicians who possess the relevant skills and knowledge:

*'Most departments at most hospitals wouldn’t even have one member of senior staff that would necessarily know what integrated care’s all about (P21TP2)*

Mentors expressed concern about a lack of clarity surrounding what would count as success in an integrated system, and suggested that more evidence is needed for measuring this beyond the edict to work together more effectively:

*'...it’s a little less clear really what the outcome measures really are beyond getting people to talk together and work together. Certainly at the population level it doesn’t quite stack up because people are going to individual surgeries from which there’s probably only a small proportion of children that actually need secondary level expertise’ (P7MP)*

Developing this theme, senior mentors in particular noted the gap between the valid aspirations and appealing rhetoric of integrated care, and the practical challenge of commissioning and designing systems that can reliably deliver it across the whole system:

*'...we can come up with these wonderful about ideas about what we think we’d like to be able to do … I think what we will have created is a cadre of people who have a better understanding of what that means, and therefore as systems change are more effective at working within these evolving systems and want to work within these evolving systems. But I actually don’t think we can say they’re ready to roll, because the system isn’t ready to roll.’ (P18MP)*

Consistent with this, suggesting that integrated care cannot be achieved without the systems that could deliver it being put in place, one clinician noted that ‘*it’s very hard to deliver integrated care without integrated care services being commissioned’ (P5MP)*. Similarly, another suggested that even when individuals or groups *do* wish to change working practices, it cannot be guaranteed that the necessary resources will be available to realise this, citing a '*lack of ability to follow through when trying to be supported by management and support structures to make change' (P12TP2).*

These quotes indicate a view which holds that integration can only be achieved via investment in the production of the necessary evidence needed for how integration can improve upon the status quo. Having identified key themes of salience emerging from our data, we will move to discussing these findings in light of the relevant literature.

## Discussion

Our study is important because it provides empirical support for critical perspectives on integrated care which can highlight the risks associated with integration that are, in our view, often obscured by the appealing rhetoric of the approach. This is not to say that we dispute the importance or value of integration; indeed, for ethical and economic reasons introduced earlier we agree that it should be pursed. However, the logic of integration entails conclusions concerning changes that will occur at accepted professional boundaries which, in the interest of patient care and supporting clinical professionals, should be addressed when designing integrated care systems. Our data supply valuable and relevant empirical data for this task.

Our findings suggest that these challenges to professional identity and autonomy may follow from attempts at integration, reflecting Baxter and Brumfitt’s (2008, p. 248) [61] observation regarding the complexity of *‘establishing professional identity and changing identity in a team context’*. A *presumption* that all care is integrated, supported by relevant education and training, is a formidable challenge because, as Lahey and Currie (2005, p. 201) [62] write, *‘in reality, the professions tend to protect their scopes of practice'.* Irvine et al (Ibid, p. 207) point out that even if all stakeholders in a care decision in principle approve of an integrated approach, ‘*agreement in principle does not automatically guarantee co-operation in practice’.*

This is significant because of what is at stake in care decisions, namely, patient wellbeing. Irrespective of one’s particular normative standpoint, any conception of the ethical treatment of patients requires clinicians to be entrusted with the right to deploy clinical judgement towards what they hold to be the right course of action in view of the specialist expertise that they have acquired. In this respect the integrity of the medical professions relies on their having clear ownership of their work. As Hall (2005, p. 191) [63] writes, '*If physicians do not...feel they are not valued for the work they are doing, they will not be enthusiastic members of a team.* Hudson (2002, p. 8) [64] also notes the importance of this for doctors:

*'Being able to identify oneself with a body of knowledge is perceived to have intrinsic worth; the professional identity … can become a valued part of individual personal identity '.*

Given that doctors are entitled to a degree of jurisdictional autonomy by virtue of having trained to acquire the specialist knowledge in that area, it is not clear what would incentivise doctors to adopt a new way of working in which this autonomy is curtailed. A considerable literature exists revealing the prestige, autonomy, and power that has historically been enjoyed by doctors, and it cannot be assumed that all doctors will find the prospect of lengthy training to acquire specialist knowledge so appealing if their authority in that domain will be encroached upon [65–67]. Indeed, Molleman et al (2008, p. 340) [68] found that doctors tended to *avoid* Multidisciplinary Team Work because it limited their clinical autonomy. Furthermore, Cameron and Lart (2003, p. 14) [69] found that successful collaboration can be undermined by ideological differences or differences in professional philosophy, which

*‘ … could lead to the emergence of distrust, professional rivalries and defensiveness between professional groups...'*

Integration is purported to ensure that clinicians can discharge their duties to patients more effectively by working across specialist boundaries and closing gaps into which patients can fall. However, doing so requires doctors to concede some of their professional autonomy and thus their professional identity. Given the personal significance of professional identity in a career such as medicine that is typically understood as not only a job but a vocation, and the dedication required to form such an identity, it is not obvious why clinicians should feel inclined to immediately embrace these changes. It may well be that clinicians *should* embrace these changes if integration will enable them to better discharge their duties to patients, but until the tension is resolved, uncertainty exists about the tractability of the theoretical ethical case for integration when its application to real-world clinician decision-making is attempted.

The inverse of professional autonomy is professional responsibility. In the context of clinicians, the responsibility is for ensuring the adequate care of their patients, and the arguments for integrated care introduced earlier contend that making care seamless will achieve this. There is evidence, including from our study, that doctors can learn from each other and successfully manage the negotiation required to make care seamless [36, 70]. Indeed, questions of responsibility that integration raises are in one respect the mirror image of those raised by the *dis*jointed care that integration is purported to fix. The danger of disjointed care caused by clearly defined boundaries of responsibility is that the gaps they create may cause harm to patients [9]. However, the risk when integrating care by overlapping domains of specialist knowledge is that opportunities are created for disputes about responsibility where it is unclear who is to be held responsible for a particular decision if harm occurs. Visse et al (2012, p. 288) [71] write:

*'...integrating services is full of ambivalence and conflicting interests...there is more to facilitating an integrated service than functionally allocating responsibilities and designing procedures to coordinate and control them … finding out ‘who should do what and why’ is often an uncertain process, filled with conflict … .'*

Looking to the future with this in mind, it is clear that the negotiation of responsibility will need to be taught as a norm of practice not only to specialists in training, but to medical undergraduates, foundation doctors, and other health professionals. Indeed, Lahey and Currie (Ibid.) [62] contend that what is needed is not *'tinkering with the boundaries’*, but '*a fundamental rethinking of core assumptions’.* An example of policy-level recognition consonant with this is HEE’s 'Shape of Caring' report (2015, p. 3) [72], which emphasises the need to expand the skills base of nurses and develop new physician assistant roles which bridge the putative boundary between the responsibilities of nurses and doctors.

A further consequence of reconfiguration towards integration is that it may partially erode boundaries between areas of clinical responsibility [73, 74], which, as Baxter and Brumfit (2008, p. 240) [61] have noted, raise *‘the danger of role confusion’* that may follow from this kind of change in approach. Their concern is that the closer one moves to the edges of one's specialism and proximity to the boundaries of adjoining domains, the more contestable the knowledge associated with it will be. This uncertainty presents a challenge. Given complications associated with scaling up integrated care from the micro- or meso- to the macro level, one might ask by what protocol are decisions about this to be made, beyond the broad principle that doctors should work collaboratively across their various specialisms.

Finally, integrated care policy guidance typically foregrounds the open-ended nature of most patients' unique situations and the need for clinicians to be flexible and comfortable with unpredictability regarding the decisions with which they will be faced. Since the need for integrated roles becomes evident through identified gaps in care which do not obviously fit into one domain or another, *'the emerging new and hybrid roles are a reflection of local requirements and, as such, are unique'.* [75]

The potential concern this raises does not count against integrated care *as such*; however, it underlines the lack of guidance about how to deliver it systemically that emerged in our study. Greater investment is needed in the production of evidence for how the envisaged reconfiguration can be realised at the level of the whole healthcare infrastructure.

### Restating the importance of integrated care training

Some clinicians might object that the risks raised in the literature and reflected in our findings are more apparent than real and do not pose a problem in practice. After all, teamwork in care is not new and clinicians and health professionals are frequently capable of agreeing on decisions and resolving ambiguities about responsibility for those decisions. However, if this objection is reasonable, then inter- and intra-professional training and education, such as in the teaching of skills in integrated care, is redundant. This conclusion is implausible.

While different clinical specialists are undoubtedly able to work together effectively, it does not follow from this that more formalised training is unnecessary. For example, whereas the nature of hospital-based secondary care may more readily offer opportunities for teamwork, the independent primary care general practitioner is more isolated [76, 77]. Barriers to making seamless the vital collaboration across primary and secondary care are thus built into the way delivery is currently structured, and inter- and intra-professional education can help to overcome this. While the quality of such education varies [78–81], Barwell et al (2013) [82], and Jacobson (2012) [83] have shown that when successful it can widen skills and improve patient safety.

Pushing this further, we can look to the analogous case of medical ethics education for trainee clinicians. As Pellegrino (1989, 1993) [84, 85] reminds us, it has been argued that formal medical ethics education is unnecessary because doctors were capable of ethical decision-making prior to the introduction of formal training in medical ethics. Historically, medical ethics training had occurred *'by osmosis'* [86] from senior clinicians, or enculturation via the ‘hidden curriculum’ [87, 88]. However, that doctors were capable of ethical practice before formal training in it became part of the standard curriculum does not undermine the legitimacy of the move to include it now.

The doctor-patient relationship has changed in recent decades, most notably in relation to a renegotiation of their respective autonomy in care decisions [42, 89]. Medicine is a public service, as McCullough (2010) [90] points out, and within certain limits the public is entitled to have its healthcare expectations met. If social norms have changed such that what it means to make a decision that is acceptable to patients requires doctors to have a more explicit understanding of medical ethics and the rights of patients to make decisions about their care, changes to norms of practice should be taught as they would be in any other area of clinical training.

Institutionally embedding training in integration throughout the medical curriculum in this way is necessary, and the policy guidance that exists for integrated care is valuable for underwriting the purported economic and ethical cases in favour of it. However, given a discrepancy between how care *ought* to be structured and delivered systemically and how it *is in fact* delivered, potential risks which will be magnified as integration is scaled up should be anticipated. This is important because of the demand that medical decision-making be *evidence-based*. As Worrall [91, 92] notes, it would be absurd to suggest that evidence is *un*important in medicine. Without an evidence base, no argument for or against can be advanced and no comparisons can be drawn with alternative approaches. Leaving aside epistemic difficulties associated with the proper definition of ‘evidence’ [93, 94], this is uncontroversial. However, without investment into research, research cannot be carried out. We suggest, therefore, that more investment is needed into scaling up integrated care research and establishing how system-wide integration might be developed, implemented, and managed.

Taking this into account it may appear that prospects are bleak for realising system-wide integration. However, this would be misleading. A recent report from the King's Fund (2018) [75] indicates that the need for evidence of this kind is gaining recognition, as ten pilot Integrated Care Systems have been established to test delivery of these processes on a larger scale at the regional level. This is welcome, but the report nevertheless reflects our argument and underlines the difficulty of reorientating the healthcare infrastructure towards system-wide integration.

Nevertheless, and despite the potential problems that we identify, the *goal* of integration is in many respects desirable. Programmes such as the one that featured in our study show how integration can be successfully implemented and so provide further evidence that integration policy need not be *only* mere rhetoric. As Hudson (2007, p. 15) [64] indicates,

*'Even though harmonious relationships may be only patchy and partial, the fact that they do exist suggests that it is time to move on from an unduly pessimistic view … The policy climate for engaging with interprofessionality has never been more propitious.'*

In summary, then, before concluding, we contend our research indicates that integration across specialist boundaries is necessary and timely. However, doing so successfully requires taking into account the implication of merging these boundaries in the name of making care seamless, namely, that domains of professional autonomy and responsibility will become increasingly negotiable and unclear. The more rigidly role boundaries are drawn, the more clearly the domain of professional autonomy for a given clinical specialism is circumscribed. Given that rigidly defined boundaries are antithetical to a project of integration – indeed, given that rigid boundaries are what integration aims to supercede – it is axiomatic that highly autonomous practice poses a challenge to integration.

## Conclusions

On the basis of our analysis of the findings in our study in light of the relevant theoretical and empirical literature, we conclude that the extant case of integration for child health examined in our study functions as a valuable exemplar for understanding challenges that are intrinsic to the project of integration across whatever domains it is attempted. The congruence between potential barriers identified by participants in the local context of child health and those that have been identified elsewhere indicate to us that much of what is true about the complexities of integration for child health is likely to hold in other contexts or care as well.

We suggest that the primary risk for ensuring that ethical obligations to individual patients are met is that practical understanding of how to construct an integrated system lags behind the theoretical arguments and appealing rhetoric in favour of it. More evidence is needed for how theory can be translated into practice in a way that improves upon the status quo. Given that evidence can only be produced via investment in its production, the duty to optimise patient care mandates such investment if it is true that successful integration *would* improve upon the structural status quo of care delivery.

Notwithstanding the valid utility-driven arguments at the macro level for integrated care, it is, as we outlined earlier, the individual patient-centred approach which situates the issue at hand in a broadly deontological and care ethics normative space. Gaps in care and understanding of individual patients’ preferences create risks and impede the ability of clinicians to discharge their duty to ensuring that patients are treated in a way that responds optimally to their interests.

Although our study found enthusiasm for integration in spite of the potential difficulties with it identified, this enthusiasm is hardly surprising given that the participants had already decided to take part in the programme. The crucial finding that the study revealed, however, was that conventional norms of professional identity and autonomy may pose a barrier to successful integration. While it may be the case that these norms should be updated, we suggest that there are reasons to take any resistance seriously.

The risk of integration is that in closing the gaps in care that result from rigidly defined clinical specialisms, patients may end up in a different kind of ‘gap’ resulting from a lack of clarity about which clinician has the autonomy and responsibility for a particular decision. If the mismatch between the elegance of theory and the complexity of practice is not taken into account, clinical ethical duties to patients may not be properly met. Irrespective of whether or not services *are* integrated, the responsibility of care must be located *somewhere,* and its location must therefore be clearly identifiable so that patients can hold clinicians to account.

Finally, on the basis of our analysis we conclude that more investment in research about how to safely scale up integrated care is needed. The next step for designing and evaluating integrated care delivery is to pilot larger scale and more complex initiatives connecting far greater numbers of hospitals, Trusts, surgeries, local authorities, and the doctors and health professionals therein. Moreover, given the current dearth of evidence there is also a need to determine the appropriate tools and approaches are for measuring integration as well as what the indicators are of its effectiveness*.* The larger and more complex the system in which integration can be achieved and delivered for the benefit of patients and health professionals, the more compelling will the evidence be that the potential obstacles outlined in this paper can be negotiated in practice and thus enshrined in policy in ways that both meet duties to patients and satisfy the professional needs of clinicians.

## Additional file

Additional file 1 Interview Schedule. (DOCX 15 kb)

## Data Availability

Relevant data are presented in the manuscript. The data that support the findings of this study are available from University College London but restrictions apply to the availability of these data, which were used under license for the current study, and so are not publicly available.

## Footnotes

1
*It is important to point out that not all patients will require their care integrated to the same degree: it is most pressing in the context of complex care needs involving numerous forms of specialist expertise.*
