# Supplementary material for: Ethical challenges of integration across primary and secondary care: a qualitative and normative analysis
Source: BMC Med Ethics. 2019 Jul 3;20:42. doi: 10.1186/s12910-019-0386-6 (PMC6610833; doi:10.1186/s12910-019-0386-6)
Supplement: Supplementary file 1 — Interview Schedule. (DOCX 15 kb) [file 12910_2019_386_MOESM1_ESM.docx]

**Appendix 1: Interview Schedule**

**PICH participants one-to-one interview schedule**

**Structure**

1. 30-45mins

**Aims of study**

The study will explore the experiences of PICH trainees and trainers across cohorts 1 and 2. The aim is to understand the impact that experience of the course has had on their understanding of the importance of and motivation to deliver integrated care in paediatrics and general practice. It will be illuminating to explore the experiences of both cohorts in order to discover what barriers and enablers are at work in relation to the delivery of integrated care. The comparison of the two cohorts will be valuable for the study because it will provide insights into intra- and interprofessional learning between medical specialities.

Your views will be fed back to Health Education England London Region, and will inform the development of subsequent intra- and interprofessional education and integrated care programmes.

We want to hear your views about your experience of participating in the PICH programme, either as a trainee or a trainer.

**Process**

1. This discussion will be recorded and analysed, looking for common themes that arise from this and other trainee and trainer interviews.
2. Transcripts will be analysed by the research team.
3. Transcripts will be anonymized and no identifying data will be presented in the report to Health Education England London region

**Consent**

Please read and sign the consent sheet and agree that everything being discussed in this interview is confidential. Do you have any questions before we start the tape?

**Background**

1. Firstly, please could you tell me whether you are from:
   1. Cohort 1 or 2?
   2. Trainee or trainer?
   3. GP or paediatrician?
2. How did you become involved in PICH?
3. Did you have any experience of integrated care or working with other medical specialities in this way prior to this program?

**The programme**

1. Please could you tell me about the PICH programme and course?
2. What do you think is / are the aim/s of PICH?
3. What aspects of the course did you feel worked well?
4. Which aspect, if any, has been the most educational / useful and why, e.g. your personal project, the CYP meetings?
5. What aspects of the program are working less well?
6. Do you think PICH has covered everything needed to enable the delivery of integrated care? If not, what was missing and could be improved upon?
7. Has participation in PICH surprised you in any way, and if so, how?
8. What have you learnt on PICH as a trainee or a trainer?

*Prompts (ensure cover of the following):*

1. *What did you learn about yourself?*
2. *About others?*
3. *About patient care?*
4. *About your organisation?*
5. How would you describe working with peers?
6. How would you describe working with trainers or trainees?

**Integrated care and working together**

1. Please reflect on working with clinicians from a different specialism, i.e. paediatrics or general practice:
   1. What have you learnt about the other specialism?
   2. What impact if any has it had on your understanding of your own?
2. Do you think interprofessional healthcare training is important, and if so why?
3. Do you think integrated care is important, and if so why?
4. Do you think there are any barriers to delivering integrated care? If so, what are they?
5. How do you think that participating in PIC H will impact on the way you work in the future?
6. What impact, if any, has PICH had on you as a clinician?

*Prompt: What have you learnt? Has PICH changed the way you work in a clinical context? If so, how?*
